# Characteristics of Highland Barley-Wheat Composite Flour and Its Effect on the Properties of Coating Batter and Deep-Fried Meat

**DOI:** 10.3390/foods12213923

**Published:** 2023-10-26

**Authors:** Jianhua Tang, Cong Xie, Wenping Chang, Zhenyang Quan, Xiangli Ding

**Affiliations:** 1 School of Tourism and Culinary Science, Yangzhou University, Yangzhou 225009, China; jhtang@yzu.edu.cn (J.T.); 212402228@stu.yzu.edu.cn (C.X.); mx120231282@stu.yzu.edu.cn (W.C.); mx120211226@yzu.edu.cn (Z.Q.); 2 Key Laboratory of Chinese Cuisine Intangible Cultural Heritage Technology Inheritance, Ministry of Culture and Tourism, Yangzhou 225009, China

**Keywords:** highland barley, composite flour, coating batter, pasting properties, oil content, deep-fried meat

## Abstract

Highland barley flour-based coating batter has rarely been reported, although highland barley flour is promising due to its high β-glucan and amylose content. In this study, highland barley flour was used to substitute 40% to 80% of wheat flour to form a highland barely-wheat composite flour used in the coating batter. The characteristics of the highland barley-wheat composite flour and its effect on the properties of coating batter and deep-fried meat were analyzed. Results showed that the composite flour significantly improved water holding capacity, oil absorbing capacity, and water solubility index. In contrast, no significant change was observed in the water absorption index or swelling power. The incorporation of highland barley flour significantly changed the pasting properties of the composite flour. Compared with the wheat flour, the viscosity and the pickup of the coating batter made with composite flour increased from 4905 Pa·s and 0.53% to more than 12,252 Pa·s and 0.63%, respectively, and its water mobility decreased. These changes were closely related to the substitution rate of highland barley flour. The composite flour significantly increased the moisture content from 27.73% to more than 33.03% and decreased the oil content of the crust from 19.15% to lower than 16.44%, respectively. It decreased L* and increased a* of the crust and decreased the hardness, adhesiveness, and springiness of the deep-fried meat. A spongy inner structure with a flatter surface was formed in all composite flour-based crusts, and the substitution rate influenced the flatness of the crust. Thus, highland barley flour could be used for batter preparation with partial substitution, enhancing the quality of deep-fried meat and acting as an oil barrier-forming ingredient for fried batter foods.

## 1. Introduction

The demand for foods made with non-wheat flour has increased in recent years. One of the motivations is that there is an increased number of individuals who want to follow a healthy diet. These health-conscious consumers relate refined wheat foods to increased mortality from lifestyle-related diseases such as diabetes, cardiovascular disease, and cancer [1]. Non-wheat flours contain high inorganic substances such as fiber, potassium, and various unique functional components. For example, the phenolic compounds in sorghum, mainly composed of phenolic acids, 3-deoxyanthocyanidins, and condensed tannins, are exceptionally unique and more abundant and diverse than other common cereals. They have potent antioxidant activity in vitro, and consumption of it was related to improved gut health and reduced risks of chronic diseases [2]. Highland barley showed a higher content of nutritional ingredients, which gives it superior antioxidant, anti-tumorigenic, and antibacterial capacities. Long-term consumption of highland barley-related products could reduce the risk of hyperlipidemia, diabetes, colon cancer, and high blood pressure [3]. Ingesting non–wheat flour habitually from the general diet in partial or complete substitution of wheat flour is an effective way to intake the functional components from non-wheat flour. Thus, diverse products made with or mainly with non-wheat flour are in great need. However, only a few staple foods and snacks, including bread, noodles, and cereal flakes, have been developed due to their poor processing properties. Batter coating is an important processing method in Asian cooking, especially in Chinese cuisine. During frying, ingredients in the coating batter form a protective film on the surface of raw materials, which prevents a direct contact between raw materials and oil, thus enhancing the fried products’ sensory quality by forming a crisp crust and a tender inside [4,5]. However, fried products are closely related to chronic diseases, such as obesity and coronary heart disease, because of their high oil content. Reducing the oil absorbed during frying is one of the fields in which most work has been performed. Currently, methods applied in the quality control of coating batters are categorized into three categories—batter formula optimization, raw materials pre-processing, and frying process improvement—among which formula optimization is the most effective [4].

Wheat flour is the typical base of coating batter due to its exceptional viscoelastic gluten, which can expand during frying, providing a desirable spongy coating. Flour/starch made from other cereals has recently been promoted to replace part of wheat flour to reduce the oil absorbed by deep-fried products. Moreover, different flours have differences in viscosity and gelatinization patterns, which affect the flavor and textures of fried products [5]. Among them, rice starch and corn starch are the two most studied. However, a higher proportion of solids or the addition of a thickener is required to achieve a suitable viscosity of the rice starch-based coating batter, and corn starch-based coating batter requires continuous mixing to avoid settling out during processing.

It is reported that a new β-glucan-rich hydrocolloid containing 32% β-glucan (C-trim 30) increased the batter pickup, decreased the moisture loss, and reduced the oil content of the sample [6]. This finding makes high β-glucan content materials a promising base for coating batter. Highland barley has a higher β-glucan content than other food crops [7]. Moreover, the higher amylose content in highland barley flour [8] enhanced its feasibility to be a non-wheat coating batter production candidate because a high amylose content was related to a low oil intake during the frying process [4]. In contrast, the utilization of β-glucan-enriched highland barley flour in coating batter was rarely reported. In this study, as a typical non-wheat flour, highland barley flour was used to substitute 40% to 80% of wheat flour in coating batter aimed at reducing the oil absorption during frying. Characteristics of the composite flour with various substitution rates were determined, and its effects on the properties of the coating batter and the final products were analyzed to enrich the variety of coating batter and deep-fried products made with non-wheat flour and to broaden the utilization of highland barley flour.

## 2. Materials and Methods

### 2.1. Materials

Wheat flour (11.7% protein, 10.4% moisture) was bought from Jinshahe Industry Co., Ltd. (Xingtai, China). Highland barley flour (59.3% starch, 10.9% protein, 8.7% moisture, 4.8% β-glucan) was provided by Rikaze Tibetan Research Food Co., Ltd. (Shigatse, Tibet, China). Corn starch, baking powder, and salt were bought from the Suguo Supermarket Co., Ltd. (Yangzhou, China).

### 2.2. Determination of Water Holding Capacity (WHC), Oil Absorbing Capacity (OAC), and Gel Hydration Properties of Composite Flour

The WHC and OAC of composite flour were determined according to the method of Beuchat and Lin, respectively [9,10]. A total of 1 g (100 mg) of composite flour was mixed with 10 mL (1.0 mL) of distilled water (edible oil) and agitated with a vortex mixer at 25 °C for 1 min to disperse flour evenly. Then, the mixture was centrifuged at 3000 rpm for 10 min at 25 °C. The supernatant was siphoned off, and the tube was upturned on filter paper to drain the water (oil). Then, the weight of the residue was recorded after 25 min of draining and was used to calculate the WHC (OAC) from the ratio of the flour sediment and the initial weight of dry flour (g moist flour/g dry flour).

The water absorption index (WAI), water-soluble index (WSI), and swelling power (SP) of the composite flour were determined with the modified method of Toyokawa, with minor modifications [11]. A total of 0.5 g of composite flour was dispersed in 10 mL of distilled water, agitated, and cooked in boiling water for 10 min, cooled in an ice water bath for 10 min, and then centrifuged at 3000 rpm at 4 °C for 10 min. The weight of the residue and the dry solids in the supernatant were determined and used to calculate WAI, WSI, and SP, respectively [12,13].

### 2.3. Determination of the Pasting Properties of Composite Flour

The pasting properties of the composite flour were determined with a rapid visco-analyzer (RVA) instrument (RVA series 4500, Perten Instruments, Waltham, MA, USA) by using the RVA Standard Pasting Method [14]. Briefly, 3.5 g of composite flour (14% moisture basis) was suspended in 25 g of distilled water, and this dispersion was stirred in aluminum canisters at 960 rpm for 10 s, followed by a constant stirring at 160 rpm until the end of the assay with the RVA temperature program. Pasting parameters including pasting temperature (PT, temperature at the moment of increase in viscosity), peak viscosity (PV, maximum hot paste viscosity), final viscosity (FV, viscosity at the end of the assay), breakdown (BD), and setback (SB) were calculated from the pasting curve with Thermocline for Windows© software (V 3.15, Perten Instruments, Sydney, Australia) [15].

### 2.4. Preparation of Coating Batters

The formulation consisted of 60 g of wheat flour, 40 g of corn starch, 2.0 g of salt, 1.0 g of baking powder, and 100 g of water (25 °C). Highland barley flour was used to substitute part of the wheat flour, and in the composited flour, its substitution ratio was controlled to be 40%, 50%, 60%, 70%, and 80%. All powder was pre-blended and mixed with water for 2 min. The coating batters were used for frying, and the determination of processing properties was performed immediately after preparation.

### 2.5. Determination of Processing Properties of the Coating Batter

Processing properties of the coating batter, including the viscosity, thermo-gravimetric properties, water mobility, and batter pickup, were determined immediately after the coating batter was prepared.

The viscosity of the prepared coating batter was measured using a rotational viscometer (NDJ-5S, Shanghai Lichen Bangxi Instrument Technology Co., Ltd., Shanghai, China). A total of 65 g of coating batter was placed in a 50 mL beaker to ensure that the rotor was submerged by the coating batter as required by the operation guide of the rotational viscometer. Measurements were conducted at 25 °C with a head speed of 12 rpm with a No. 3 rotor. Measured values were recorded and reported as Pa·s [16].

Thermogravimetric analysis (TGA) on the coating batter was carried out immediately after the coating batter was prepared with a thermogravimetric method and simultaneously differential thermal analysis (TGA/SDTA) (Pyris 1 TGA, PerkinElmer, Waltham, MA, USA). Approximately 10 mg of coating batter was used for each determination, and each sample was heated from 25 °C to 200 °C at a heating rate of 10 °C/min in a nitrogen (20 mL/min) atmosphere.

Water mobility of the coating batter was determined with low-field nuclear magnetic resonance (LF-NMR) relaxation measurements using an NMI20-060 H-I-25 mm (NMR, Suzhou Niumai Analytical Instrument Ltd., Suzhou, China) as described by Cao et al. [17], with slight modification. Transverse relaxation time (T2) was measured using the quadrupole echo CPMG (Q-CPMG) sequence. The coating batter (~5 g) was placed in a sample stage and inserted in the NMR probe. Parameters were set as follows: signal frequency (21 MHz), signal frequency offset (440,084.47 Hz), 90-degree pulse width (6.20 µS), 180-degree pulse width (12.40 µs), the frequency range of the signal received by the receiver (250 kHz), control parameters of sampling starting point (0.00200 ms), sampling frequency (250), simulation gain (20.0 db), digital receiver gain (3), and number of echoes (10,000). The NMR data were analyzed using the method described in previous studies [17].

Batter pickup was calculated with the mass of meat before and after coating with the following formula:

Batter pickup (%) = (m_A_ − m_B_)/m_B_ × 100 (1)

where m_B_ and m_A_ are the mass of the meat before and after batter coating, respectively.

### 2.6. Preparation of Fried Battered Meat

Frozen pork chops were cut into sheets (3 cm × 3 cm × 0.5 cm) and thawed at room temperature. Drop loss from the meat was removed from the surface with kitchen paper. Then, they were immersed in the coating batter for 10 s, drained for 10 s, and deep-fat fried at 200 ± 5 °C for 3 min with a commercial fryer (EF-101, Longling Commercial Equipment Hotel Co. Ltd., Foshan, China) with soya bean oil. Deep-fried samples were cooled at room temperature for 10 min before analysis, during which excessive oil was drained.

### 2.7. Properties of Deep-Fried Meat

The crust peeled off the meat was used to analyze the moisture and oil content of the crust using a direct drying method at 105 °C and according to the AOAC official methods 960.39 [18,19].

The color profile of the crust of the deep-fried meat was determined with an automatic colorimeter (ACD1-2000, Chengtaike, Beijing, China). L*, a*, and b* were recorded.

The textural properties of the meat in the deep-fried meat were measured using a TA analyzer (TA. TOUCH, Bosin Tech, Shanghai, China) with a TA/75 probe at a deformation rate of 75%, a load cell weight of 20 kg, and a test speed of 2 mm/s. The meat was cut into a round shape with a 1.1 cm diameter with a mold before determination. Hardness, adhesiveness, springiness, chewiness, cohesiveness, and resilience were recorded.

### 2.8. Microstructure of Deep-Fried Meat

Deep-fried meat was freeze-dried, sliced, and sputter-coated with a gold–palladium alloy, and the microstructure of the crust and the interface of the crust and the meat were observed with SEM (S-4800 II, Hitachi, Tokyo, Japan) under vacuum and at an accelerating voltage of 5 kV, respectively. Representative micrographs were taken for each sample at a magnification of ×100 and ×50 for the crust and the interface of the crust and the meat, respectively.

### 2.9. Statistical Analysis

SPSS Statistics 21.0 (SPSS Inc., Chicago, IL, USA) was used to analyze the significance of the impact factor data using one-way analysis of variance (ANOVA). All statistical analyses were performed with Duncan’s post hoc test, and a significance level of *p* < 0.05 was used. The Origin 2022 (OriginLab Corporation, Northampton, MA, USA) was used in the data treating and diagram making.

## 3. Results and Discussion

### 3.1. WHC, OAC, and Gel Hydration Properties of Composited Flour

The composited flour’s hydration and gel hydration properties were determined, and the results are shown in Table 1. Results showed that the incorporation of highland barley flour in the composite flour significantly changed the WHC and WSI, respectively. A significant increase in OAC was only found when more than a 60% substitution rate was used. In contrast, no significant difference was observed in WAI and SP. Increased water retaining ability of the coating batter was also observed with C-trim 30 [6]. It is reported that the WHC of the dry ingredients in the batter shows a decisive influence on its viscosity [20], thus influencing batter pickup, batter-retaining capability, and the final quality of deep-fried products. Although composite flour with 40% highland barley flour substitution showed a significantly higher WHC than wheat flour, significant changes were only found among samples once the substitution rate was increased to 80%. This implied that the WHC is mainly determined by the type of flour. Moreover, the gel network formed by the high content of β-glucan and arabinoxylan in highland barley flour is the main reason for the increased WHC. The WHC was influenced both by the β-glucan and arabinoxylan content and by the crosslinking density of the gel network formed [21]. When the degree of crosslinking is higher than the optimum, swelling is impeded, and this is reflected by a constant or decreased WHC. The increased OAC at a 70% and 80% substitution would help facilitate the penetration of oil during frying. SP reflects the water absorption characteristics of starch during gelatinization and the WHC of the paste after gelatinization [22]. The WSI and SP reflect the interaction between starch and water [23]. The increased WSI implied that the incorporation of highland barley flour improved the interaction between flour and water due to its higher small granular starch and β-glucan content [24]. SP reflects the water-absorbing and holding capacity during gelatinization [25]. It is reported that highland barley starch has higher solubility and SP than wheat starch at the same temperature, implying that it enhances the intensity of the starch–water interaction [22]. Thus, the non-significant difference observed in the SP between wheat flour and composite flour implied that ingredients in the flour other than starch would also influence the SP of the flour, and that the non-significantly changed SP of the composite would play its role by creating an effective barrier against oil in fried, battered products.

### 3.2. Pasting Properties of Composited Flour

**Figure 1:**
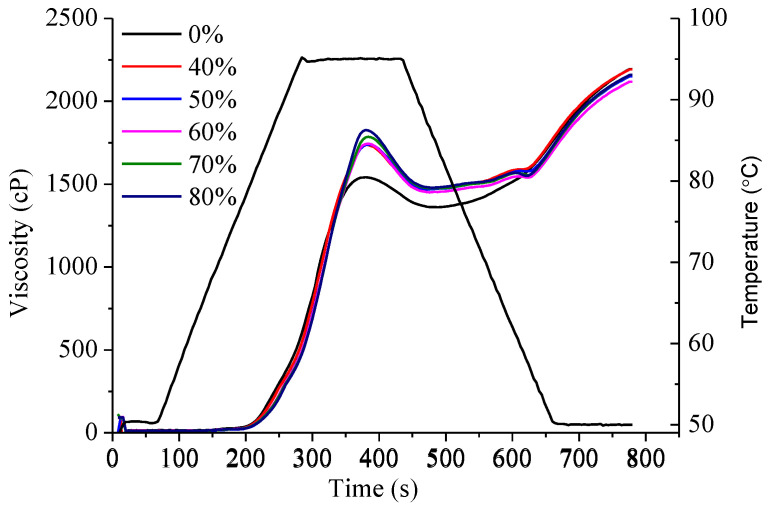
Pasting curves of highland barley-wheat flour with various highland barley flour substitution rates.

Determination of the pasting properties of starch-based paste is helpful in cereal blend formulations and in the feasibility of its application assessments [26]. Thus, RVA was used to mimic gelatinization during frying, and the effect of the incorporation of highland barley flour in the composite flour on the pasting properties was analyzed with PT, PV, FV, BD, and SB. The incorporation of highland barley flour significantly changed the pasting curve of the composite flour, as shown in Figure 1. It increased the PT, PV, and BD, whereas it decreased SB and FV, respectively. Similar results were also found when wheat flour was substituted with barley, rye, millet, or sorghum in the form of whole grains (individually, and in blends) [26]. Bello-Perez et al. found that the gelatinization temperature of highland barley starch is lower than that of wheat starch [27]. Thus, the increased PT of the composite flour would be caused by the lower free water in composite flour. PV indicates the WHC of the starch or mixture. Although lower PV values of highland barley starch than wheat starch were reported before [27], an increased peak viscosity was observed in the composite flour in this study. This is in accordance with the WHC determined in the hydration properties discussed above. Speculations can be made that the increased PV of the composite flour was not caused by the change in the starch composition in the composite flour but by the changes in water–flour interaction. FV indicates the viscous paste-forming ability of the starch after cooking and cooling. Decreased FV of the composite flour implied that highland barley flour would decrease the viscosity of the coating batter after gelatinization, and this would benefit the sensory acceptance of the deep-fried meat by improving its crisp texture. The BD value of the composite flour is greater than that of the wheat flour. This indicated that composite flour features a lower degree of swelling, and swollen starch in it is easily broken. SB reflects the re-association between starch molecules during cooling, and it has been correlated with the texture of the final products. Decreased SB of the composite flour indicates a low rate of starch retrogradation [26], and it is meaningful for a prolonged shelf-life of the deep-fried products.

### 3.3. Viscosity of Batters

**Figure 2:**
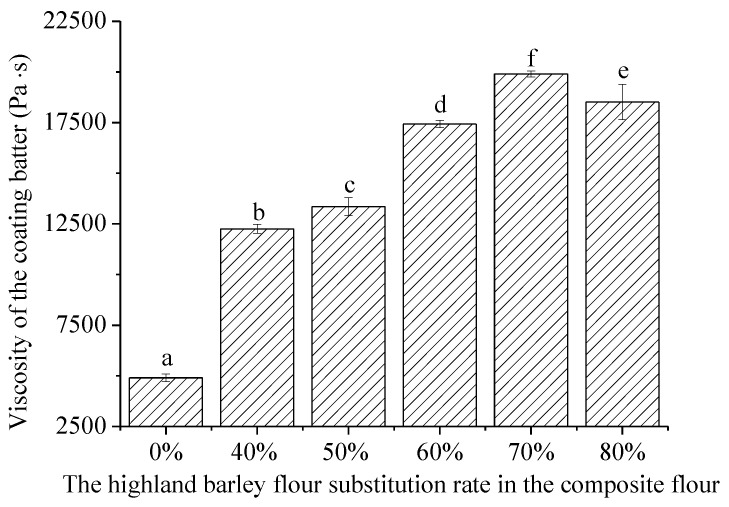
Effect of highland barley flour substitution rate on the viscosity of the coating batter made with composite flour. ^a–f^ The values with different lower-case letters are significantly different (*p* < 0.05).

The effect of highland barley flour substitution on the viscosity of the coating batter is shown in Figure 2. It can be seen that the incorporation of highland barley flour had a significant influence on the viscosity of the coating batter. The viscosity of the coating batter increased 2–3 times, with the substitution rate increasing from 40% to 70%. The high content of damaged starch [28] and dietary fiber (12.8–17.2%) of highland barley flour, especially its β-glucan, has long been thought to be the main reason for the increased viscosity because damaged starch can absorb more water than native granules [29]. Moreover, the higher damaged starch content in highland barley flour may affect the viscosity of the flour-water mixture both with and without heating [4,8]. Highland barley starch has higher solubility and swelling power than wheat starch at the same temperature. It is well-accepted that the batter coating performance can be predicted by its viscosity [30], as the viscosity could be closely correlated with the batter pickup and the thickness of the crust formed after deep frying [31]. Enough viscosity provides appropriate adhesiveness of the coating batter to the food substrate that needs to be coated during coating. However, excessive viscosity leads to reduced processing properties and stability of the coating batter, which should be taken into consideration in industrial applications.

### 3.4. Batter Pickup

**Figure 3:**
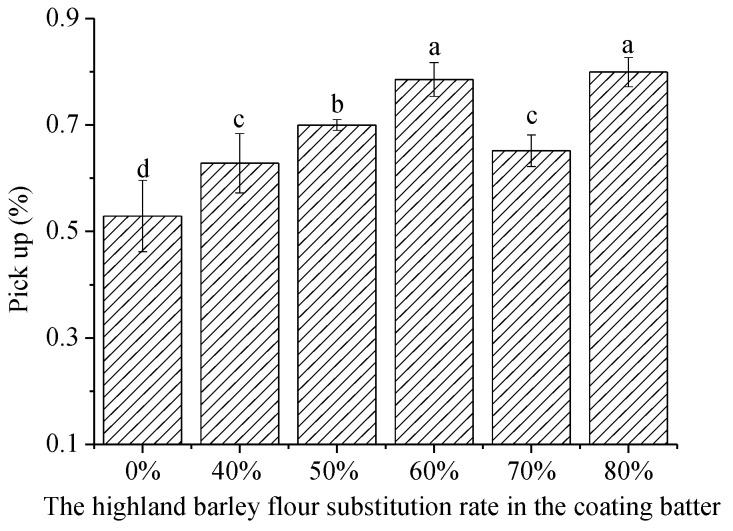
Effect of highland barley flour substitution on the pickup of the coating batter. ^a–d^ The values with different lower-case letters are significantly different (*p* < 0.05).

Batter pickup depends mainly on its viscosity. That is, the higher the viscosity, the higher the pickup [32], as proved by C-trim 30-containing batters studied by Lee and Inglett [6]. In this study, coating batter made with highland barley-wheat composite flour all showed higher pickup as compared to coating batter made with wheat flour only (Figure 3), implying that the incorporation of highland barley flour helps it tenaciously adhere to the meat during processing. To be specified, a decreased pickup and no significant change were observed when the highland barley flour substitution rate was increased from 60% to 70% and 80%, respectively. Thus, excessive viscosity would result in increased dripping of the coating batter during draining due to its own weight, reflected by decreased pickup. A high pickup is related to the formation of a crispy crust after deep frying and a high product yield from the perspective of industrialization [6].

### 3.5. Water Mobility of the Coating Batter

**Figure 4:**
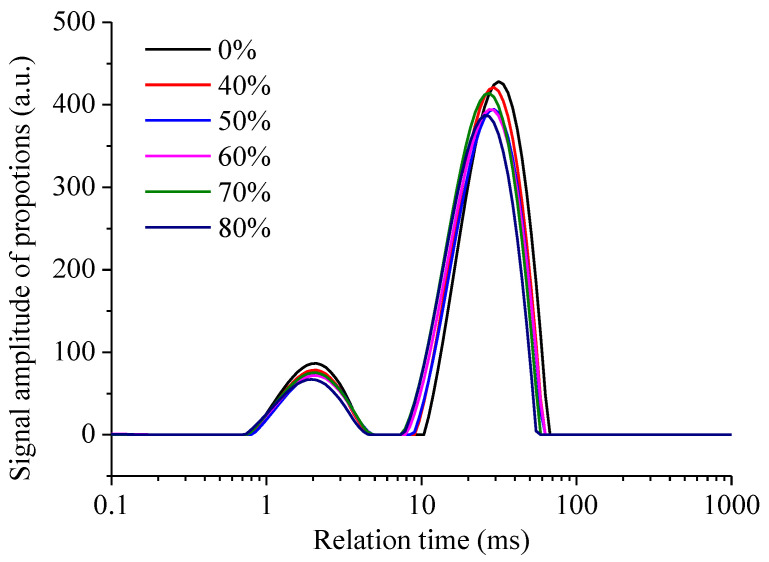
Effect of highland barley flour substitution rate on the water mobility of the coating batter.

Water mobility of the coating batter was determined with LF-NMR. It can be seen from Figure 4 that the incorporation of highland barley flour in the composite flour significantly changed the water mobility of the batter, as an evident shift of the curves towards the shorter relation time can be observed. That is, in general, highland barley flour in composite flour decreased the water mobility of the coating batter. Three components with different mobilities were observed in the coating batter, marked as T21 (0.092–1.956 ms, the bound water), T_22_ (1.956–25.529 ms, immobilized water), and T_23_ (25.529–62.950 ms, free water). T_21_ cannot be clearly seen from the curve because it only takes up 0.034% to 0.150% of the total peak area. The small T_21_ showed that water hydration in the coating batter was restricted during the mixing of the flour and water. The peak area ratio of T_22_ decreased from 15.827% to 14.526% when 40% highland barley flour was used as the substitution and then decreased to 13.649% when the substitution rate was further increased to 80%. In contrast, the peak area ratio of T_22_ decreased from 15.827% to 14.526% when 40% highland barley flour was used for the substitution and decreased to 13.649% when the substitution rate was further increased to 80%. The shift of the curves and the change in the peak area indicated that the incorporation of highland barley decreased the water mobility in the coating batter, and this influence was closely related to the substitution rate of highland barley flour. The water replacement mechanism is one of the three proposed mechanisms of oil absorption during deep-fat frying [33]. It is commonly accepted that the oil uptake is largely determined by the moisture content in the food [34]. During deep frying, water evaporates from the batter rapidly, leaves the surface pores and voids, and these areas could be filled with oil [6]. Thus, the oil uptake during frying is directly correlated to the water evaporation, and the moisture content of the flour base has been highly correlated with the textural characteristics, oil absorption, appearance, and overall acceptability [4]. In this study, a conclusion can be made that, instead of the moisture content of the coating batter, water mobility plays a more important role. Decreased water mobility in composite flour would impede water evaporation during deep frying and then decrease the oil content of the deep-fried meat.

### 3.6. TGA-SDTA

**Figure 5:**
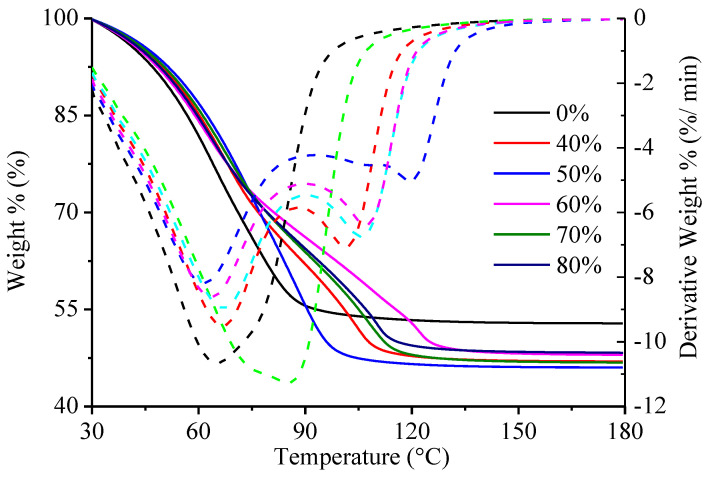
Effect of highland barley flour substitution rate on the water loss of the coating batter.

TGA-SDTA on the coating batter was conducted to monitor water loss by mimicking the heating process during deep frying. Results are shown in Figure 5. It can be seen from the TGA curves (solid line) that the incorporation of highland barley flour in the composite flour significantly changed the trend of weight loss and water retention after heating. Differences in the result of SDTA confirmed that the derivative weight changed variously among samples. To be specific, the change of the derivative weight of the composite flour-based coating batter occurred with more complexity because it peaked twice, in comparison to a unique peak calculated in the wheat flour-based coating batter. A significant weight loss was observed at around 70 °C and is shown as the first peak of the derivative weight in the dashed line in Figure 5. The lower derivative weight of the composite flour-based coating batter, when the temperature is higher than 90 °C, indicated that water evaporated from the composite flour-based coating batter more slowly than that from wheat flour-based coating batter. This may have occurred due to an increased viscosity and decreased water mobility, which were discussed above. Decreased weight retained in the composite flour-based batter was observed from the curves of weight change. A higher amount of water loss is commonly related to an increased oil uptake [34]. Thus, the incorporation of highland barley flour would result in an increased oil content in deep-fried meat.

### 3.7. Moisture and Oil Content

Moisture and oil content of the crust after deep frying was determined, and is listed in Table 2. Results showed that incorporating highland barley flour in the composite flour significantly increased the moisture and decreased the oil content in the crust, respectively. In contrast to the decreased weight retained during programmed heating with TGA, increased moisture content was observed in the composited flour-based crust. This implied that TGA could be used to reflect the trend of water evaporation during heating; however, it cannot be used to predict how much water was retained after heating. The composite of the flour mainly influenced the moisture content in the crust. Water replacement mechanisms have been suggested to elucidate oil absorption mechanisms when oil is used as the heat transfer medium during deep-fat frying. With this mechanism, oil uptake was mainly contributed by the oil absorbed on the surface of raw materials and the oil absorbed by comparatively big pores in the fried food formed as a result of moisture loss [35]. Changes in the characteristics of the coating batter would greatly influence the amount of oil uptake during frying because moisture loss and oil absorption is a surface phenomenon. The insignificantly changed OAC of composite flour until a 70% substitution rate would be one of the reasons for the decreased oil content of the crust. Moreover, a strong relationship was found between moisture loss and oil absorption when polysaccharides were used in coated products during frying, and this effect was ascribed to the thermo-gelling properties against moisture loss of these polysaccharides, which led to an increased WHC [35]. Thus, the high WHC of the composite flour with a 40% to 80% substitution would be another reason for the increased moisture and decreased oil content. Moreover, the pickup of the coating batter also plays its role because the batter retained on the meat’s surface determines the coating’s thickness, which influences the heat transfer from the oil to the batter during frying. Reducing the quantity of oil absorbed during frying is one of the fields in which most work has been performed on coating batters’ development in recent years [4]. Reduced oil content was conferred by the addition of gluten and hydroxypropyl methylcellulose [18,36]. Similar to the moisture content, the oil content does not decrease along with the increase in the substitution rate of highland barley flour. The lowest oil content was reached when a 60% substitution was used. The high oil content in fried foods is caused by the oil absorbed on the surface of raw materials during deep oil frying. Thus, the significantly increased OAC of the composite flour would be the reason for the increased oil content of the crust when more than 60% wheat flour was substituted by highland barley flour [35]. The decreased oil content with the incorporation of highland barley flour in the coating batter implied that highland barley flour is an outstanding ingredient that can be widely used in the development of coating batters as a natural material.

### 3.8. Color of the Crust and Texture Properties of Deep-Fried Meat

In general, the incorporation of highland barley flour in composite flour significantly changed the L* and a* of the crust of fried meat, as shown in Table 3. Moreover, its influence on b* was not significant. Significantly decreased values of L* and increased a* were translated into a more intense golden color, which was perfectly acceptable in all of the batter-coated products. A similar change was also reported when various ingredients were added to a batter coating for fried seafood [36]. When highland barley flour was used in yellow alkaline noodles, it decreased the L*, whereas no significant influence was observed on L* and a*. However, a significant increase in b* was observed when it was added to white salted noodles. Thus, the influence of highland barley flour on the color parameters of the final products varied according to the type of product. Moreover, changes in L* and a* were not linearly along with the highland barley flour substitution rate. That is because the color of the crust was not only influenced by the ingredients of the coating batter or by the thickness of the coating batter, that is, the pickup of the coating batter.

Except for the resilience and cohesiveness, all textural properties of the meat were significantly changed with composite flour in the coating batter. The hardness of cooked meat is crucial to its overall acceptance. Thus, it can be used to predict sensory textural acceptance [37]. The lower the hardness, the higher the juiciness and tenderness. The significantly decreased hardness implied that the coating batter formed by composite flour helps prevent water loss during frying. The decrease in adhesiveness was closely related to the substitution. To be specific, a decreased springiness was observed in all composite flour-based samples except for samples with a 60% substitution. Further studies are needed to interpret the underlying mechanisms.

### 3.9. Microstructure

**Figure 6:**
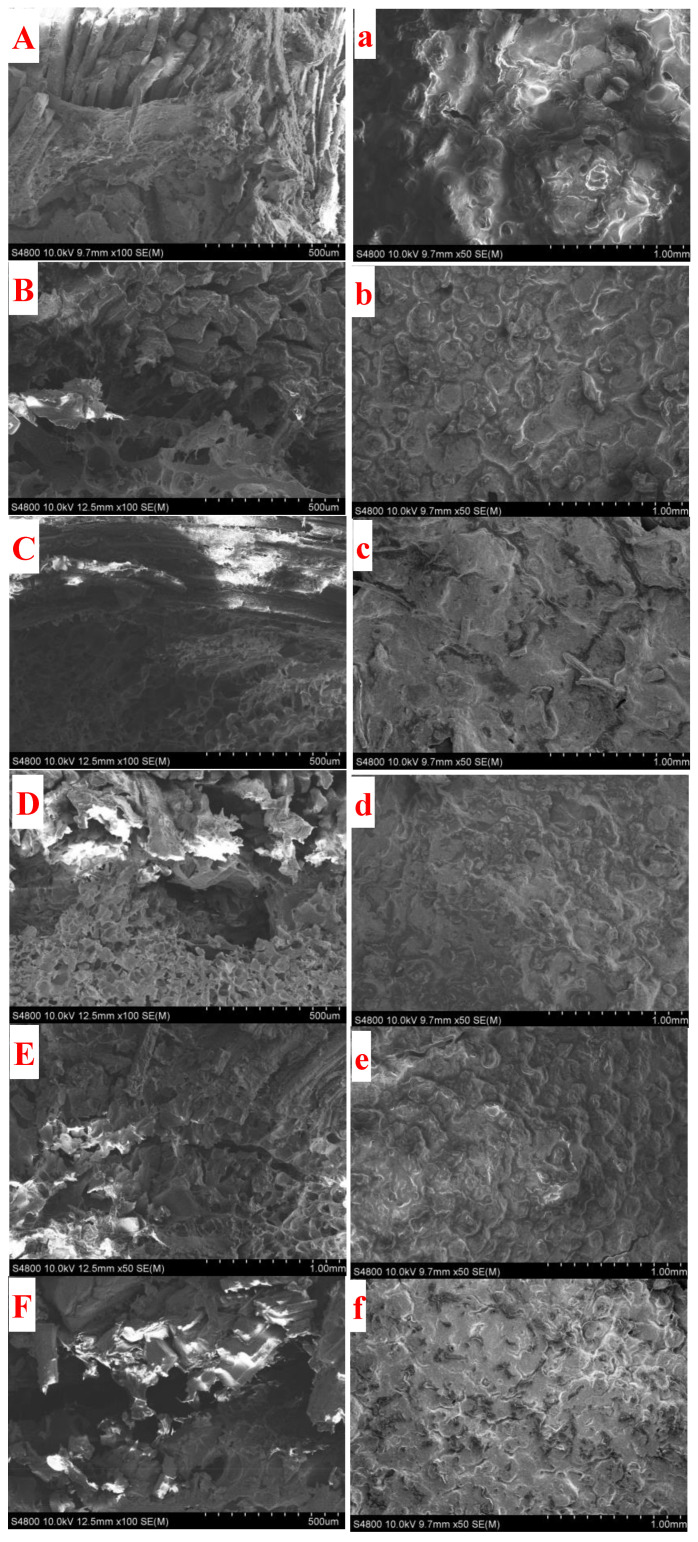
Microstructure of deep-fried coating batter at the interface of the meat and crust (**A**–**F**) and the surface of the crust (**a**–**f**). From (**A**,**a**–**F**,**f**), the highland barley flour substitution rate was changed from 0 to 40%, 50%, 60%, 70%, and 80%, respectively.

Transport phenomena in deep-fried foods depend strongly on their structure [38]. SEM is commonly used to reveal fried products’ internal structure and surface appearance. The microstructure of deep-fried coating batter was observed with SEM on the interface of the meat and crust, and results were shown in Figure 6A–F. The wheat flour-based crust is closely adhesive on the meat, whereas obvious gaps can be observed in the composite flour-based crust and meat. This is because the peak viscosity of the composite flour-based coating batter is much higher than that of the wheat flour, and the starch film formed was pushed out as a whole by the vapor from the inner meat during heating. Moreover, the microstructure of the cooked batter made with composite flour also changed significantly. In contrast to the condensed structure in the wheat flour-based crust, a spongy structure was observed in all composite flour-based crusts. This spongy structure would also be attributed to the high water absorption and gel formation capacity of the highland barley flour. A similar spongy crust was observed in the crust of deep-fried chicken meat when whey protein was added [39]. This was ascribed to the continuous protein matrix formed by denatured protein and gelatinized starch during frying. Importantly, oil absorption is a surface-related phenomenon [40]. Then, the roughness of the crust was also observed with a feature of theres being holes and pores which formed on the surface. Images in Figure 6a–f implied that highland barley flour incorporation helps to form a flatter surface of the crust. Moreover, the substitution rate influenced the flatness of the crust. It is reported that the surface of the coating was full of air cells with numerous vented holes, allowing oil to penetrate during frying and cooling in the microstructure of fried wheat flour-based batters [38]. In comparison, vented holes are rarely seen in this study. This may be because the viscosity of the batter that was used was quite different. When 80% highland barley flour was incorporated, noticeable pores were seen, as illustrated in Figure 2f. This implied that too much highland barley would have a detrimental effect on the crust.

## 4. Conclusions

As one of the non-wheat flours, highland barley flour is a promising base for coating batter due to its high β-glucan and amylose content. Highland barley-wheat composite flour was produced by partially substituting wheat flour with highland barley flour to explore its possibility of being utilized as a coating batter. Characteristics of highland barley-wheat composite flour and its effect on the properties of coating batter and deep-fried meat were analyzed. Results showed that the incorporation of highland barley flour significantly influenced and increased the WHC, OAC, and WSI and significantly changed the pasting properties of the composite flour. Moreover, it increased the viscosity and the pickup, and decreased the water mobility of the coating batter. The composite flour significantly increased the moisture content and decreased the oil content of the crust, decreased L* and increased a* of the crust, decreased hardness, adhesiveness, and springiness of the deep-fried meat, and helped to form a spongy inner structure with a flatter surface in composite flour-based crusts. This influence was closely related to the substitution rate of highland barley flour. Thus, highland barley flour can be used in coating batter preparation to obtain deep-fried products with a lower oil content and improved textural properties. Consequently, the composite flour’s high WHC and pasting viscosity limits its substitution rate because it leads to excessive viscosity, decreased pickup, and increased processing difficulty. Nevertheless, it is prospective in rice flour-based coating batter to provide it with a suitable viscosity, which commonly requires a greater proportion of solids or the addition of a thickener to achieve this.

## Figures and Tables

**Table 1:** WHC, OAC, and gel hydration properties of composited flour.

Samples	WHC	OAC	Gel Hydration Properties		
			WAI	WSI	SP
0%	1.74 ± 0.01 ^a^	1.73 ± 0.03 ^a^	8.90 ± 0.45 ^a^	4.79 ± 0.16 ^a^	9.35 ± 0.46 ^a^
40%	1.81 ± 0.00 ^b^	1.78 ± 0.04 ^ab^	8.91 ± 0.23 ^a^	6.67 ± 0.55 ^bc^	9.55 ± 0.19 ^a^
50%	1.83 ± 0.00 ^b^	1.78 ± 0.00 ^ab^	8.98 ± 0.16 ^a^	6.62 ± 0.37 ^bc^	9.62 ± 0.13 ^a^
60%	1.84 ± 0.00 ^b^	1.79 ± 0.01 ^ab^	9.17 ± 0.33 ^a^	6.30 ± 0.40 ^b^	9.78 ± 0.39 ^a^
70%	1.85 ± 0.00 ^b^	1.83 ± 0.01 ^b^	8.71 ± 0.08 ^a^	7.30 ± 0.14 ^c^	9.39 ± 0.07 ^a^
80%	1.90 ± 0.00 ^c^	1.84 ± 0.01 ^b^	8.78 ± 0.02 ^a^	6.24 ± 0.17 ^b^	9.37 ± 0.04 ^a^

^a–c^ Values in the same column followed by different superscript letters are significantly different (*p* < 0.05). WHC, water holding capacity; OAC, oil absorbing capacity; WAI, water absorption index; WSI, water solubility index; SP, swelling power.

**Table 2:** Moisture and oil content of the crust after deep frying.

Sample	Moisture Content	Oil Content (%)
0%	27.73 ± 0.61% ^a^	19.15 ± 1.06 ^a^
40%	41.29 ± 4.60% ^cd^	14.91 ± 0.96 ^b^
50%	33.03 ± 0.49% ^ab^	16.44 ± 1.98 ^ab^
60%	37.95 ± 0.90% ^bc^	10.32 ± 0.09 ^d^
70%	36.45 ± 2.62% ^bc^	13.97 ± 1.72 ^bc^
80%	45.66 ± 0.25% ^d^	11.37 ± 0.35 ^cd^

^a–d^ Values in the same row followed by different superscript letters are significantly different (*p* < 0.05).

**Table 3:** Color of the crust and textural properties of deep-fried meat coated with composited flour-based coating batter at various substitution rates.

Sample	0%	40%	50%	60%	70%	80%
L*	63.77 ± 1.86 ^b^	63.94 ± 2.22 ^b^	65.18 ± 3.03 ^b^	65.56 ± 2.99 ^b^	59.56 ± 2.77 ^a^	56.67 ± 2.94 ^a^
a*	4.99 ± 0.77 ^a^	6.64 ± 1.62 ^c^	5.28 ± 0.91 ^ab^	5.25 ± 0.30 ^ab^	6.39 ± 1.04 ^bc^	6.58 ± 0.74 ^c^
b*	19.05 ± 1.94 ^ab^	20.74 ± 3.07 ^ab^	20.70 ± 1.01 ^ab^	18.82 ± 0.98 ^a^	21.21 ± 1.57 ^ab^	19.99 ± 1.00 ^ab^
Hardness (N)	105.10 ± 5.28 ^d^	81.09 ± 2.89 ^c^	82.99 ± 2.42 ^c^	86.56 ± 4.66 ^c^	72.28 ± 6.19 ^b^	64.87 ± 6.27 ^a^
Adhesiveness (N·sec)	−0.10 ± 0.12 ^c^	−0.81 ± 0.10 ^b^	−0.80 ± 0.10 ^b^	−0.77 ± 0.30 ^b^	−0.71 ± 0.39 ^b^	−1.25 ± 0.41 ^a^
Springiness	0.78 ± 0.02 ^bc^	0.72 ± 0.05 ^b^	0.70 ± 0.07 ^a^	0.81 ± 0.04 ^c^	0.70 ± 0.03 ^a^	0.66 ± 0.06 ^a^
Cohesiveness	0.77 ± 0.02 ^c^	0.74 ± 0.05 ^bc^	0.71 ± 0.01 ^abc^	0.74 ± 0.05 ^bc^	0.67 ± 0.04 ^a^	0.68 ± 0.06 ^ab^
Resilience	0.29 ± 0.02 ^a^	0.30 ± 0.03 ^a^	0.27 ± 0.01 ^a^	0.29 ± 0.04 ^a^	0.27 ± 0.05 ^a^	0.27 ± 0.03 ^a^

^a–d^ Values in the same row followed by different superscript letters are significantly different (*p* < 0.05).

## Data Availability

All data are available from the corresponding author upon reasonable request.
